# Nucleic Acid-Based Approaches for Tumor Therapy

**DOI:** 10.3390/cells9092061

**Published:** 2020-09-09

**Authors:** Simone Hager, Frederic Julien Fittler, Ernst Wagner, Matthias Bros

**Affiliations:** 1 Department of Chemistry and Pharmacy, Ludwig-Maximilians-University (LMU), 81377 Munich, Germany; ernst.wagner@cup.uni-muenchen.de; 2 Department of Dermatology, University Medical Center, 55131 Mainz, Germany; ffittler@students.uni-mainz.de

**Keywords:** nucleic acids, nanoparticle, transgene, antigen, adjuvant, dendritic cell, tumor, immunotherapy

## Abstract

Within the last decade, the introduction of checkpoint inhibitors proposed to boost the patients’ anti-tumor immune response has proven the efficacy of immunotherapeutic approaches for tumor therapy. Furthermore, especially in the context of the development of biocompatible, cell type targeting nano-carriers, nucleic acid-based drugs aimed to initiate and to enhance anti-tumor responses have come of age. This review intends to provide a comprehensive overview of the current state of the therapeutic use of nucleic acids for cancer treatment on various levels, comprising (i) mRNA and DNA-based vaccines to be expressed by antigen presenting cells evoking sustained anti-tumor T cell responses, (ii) molecular adjuvants, (iii) strategies to inhibit/reprogram tumor-induced regulatory immune cells e.g., by RNA interference (RNAi), (iv) genetically tailored T cells and natural killer cells to directly recognize tumor antigens, and (v) killing of tumor cells, and reprograming of constituents of the tumor microenvironment by gene transfer and RNAi. Aside from further improvements of individual nucleic acid-based drugs, the major perspective for successful cancer therapy will be combination treatments employing conventional regimens as well as immunotherapeutics like checkpoint inhibitors and nucleic acid-based drugs, each acting on several levels to adequately counter-act tumor immune evasion.

## 1. Introduction

Cancer is a serious and life-threatening disease with increasing incidence in today’s world [1,2,3,4,5]. Depending on the tumor type, stage, and location, cancer therapy can be very challenging. Conventional treatments (surgery, chemotherapy, and irradiation) are often inefficient, resulting in recurrence and even death. The main reasons for therapy failure are chemoresistance as well as metastasis [6,7]. Moreover, the patients often suffer from severe side-effects [8]. In the last 20–30 years, however, cancer treatment regimens have changed remarkably, based on the gained knowledge about molecular biology as well as tumor pathobiology and pathophysiology [9,10,11]. As a consequence of a better understanding of the tumor as a heterogeneous tissue with different types of cells, new strategies for cancer therapy have been developed, which are also applicable in combination with classical therapies [12,13,14,15,16,17,18,19,20,21,22,23,24]. However, still only a limited number of patients respond to the already approved immunotherapies, and toxicity as well as induction of resistance towards treatment are often a problem [25,26,27,28,29]. Nanotechnology-based strategies, and in particular therapeutic nucleic acids, as well as combined immunotherapies may improve the therapeutic outcome in more patients for a broad range of tumors, even in late stage. In this regard, nucleic acid-based immunotherapeutic approaches have received growing interest [24,30,31].

**Figure 1:**
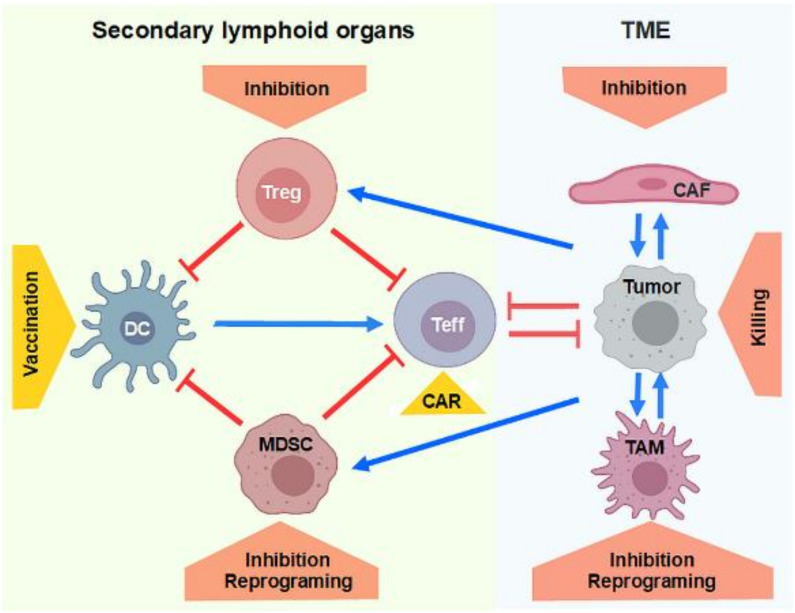
Nucleic acid-based strategies for tumor therapy. Vaccination of dendritic cells (DC) aims to induce tumor-specific effector T cells (Teff), which in turn kill tumor cells. Regulatory immune cells, regulatory T cells (Treg) and myeloid-derived suppressor cells (MDSC), are induced by the tumor and other cells of the tumor microenvironment (TEM) and inhibit both DC and Teff. The expansion and suppressive activity of Treg/MDSC can be inhibited by RNA interference (RNAi) and MDSC may be reprogramed to yield antigen presenting cells by applying nucleic acid-based stimuli. Further, T cells can be transfected/transduced with chimeric antigen receptors (CAR) to gain tumor specificity. Teff are inhibited by factors within the TME. Tumor-specific delivery of nucleic acids (gene-coding or conferring RNAi) is aimed to induce apoptosis in tumor cells, and to inhibit or reprogram accessory cells within the TME, tumor-associated macrophages (TAM), and cancer-associated fibroblasts (CAF).

This review aims to present a comprehensive overview of the current state of nucleic acid-based anti-tumor therapeutics, and associated optimization strategies. As depicted in Figure 1, such strategies aim (i) to deliver tumor-related antigen plus adjuvant to antigen presenting cells (APC) like dendritic cells (DC) that induce tumor-specific immune responses, (ii) to either deplete or reprogram tumor-induced/expanded immunoregulatory cell types, especially regulatory T cells (Treg) and myeloid-derived suppressor cells (MDSC), which collectively inhibit the induction of adaptive immune reactions in the periphery, (iii) to generate tumor-specific T cells and natural killer (NK) cells by genetic introduction of synthetic antigen receptors, termed CARs (chimeric antigen receptors), and (iv) at the tumor site itself to yield direct tumor cell killing, and to inhibit the tumor-promoting function of the tumor microenvironment (TEM). It is worth mentioning that the first clinical trial ever using in vivo gene transfer was conducted by Nabel et al. in 1993 with an intratumorally applied liposomal formulation of immunotherapeutic DNA encoding for HLA (human leukocyte antigen)-B7 [32].

## 2. Nucleic Acid-Based Strategies to Induce Adaptive Anti-Tumor Responses

In the last decades, the potential to exploit the patients´ immune system to induce and shape anti-tumor responses has gained increasing interest [33]. The induction of tumor antigen-specific adaptive immune responses requires co-delivery of the antigen and of an immunostimulatory compound to evoke activation of a professional antigen presenting cell (APC) [34]. In this regard, DC that are considered the most potent APC population at stimulated state are in the focus of interest [35]. In conventional vaccination approaches, the antigen is applied as a peptide/protein in combination with a structurally different adjuvant that specifically triggers a danger receptor expressed by DC (and other APC) [36]. According vaccination approaches need to overcome several obstacles like (i) unwanted uncoupling of antigen and adjuvant in vivo, which may contribute to unwanted immune reactions, (ii) binding/uptake of the vaccine by non-APC, including regulatory immune cells like tumor-associated macrophages (TAM) and tumor-induced myeloid-derived suppressor cells (MDSC), which may result in the induction of tumor immune tolerance, and (iii) limited presentation of the exogenous antigen via major histocompatibility complex class I (MHCI), yielding limited activation of CD8^+^ T cells, and thereby insufficient induction of cytotoxic tumor lymphocytes (CTL). As outlined in the following, nucleic acids encoding for antigens (plasmid DNA (pDNA) or mRNA) and nucleic acid-based adjuvants, especially when encapsulated in APC-targeting nanoparticles (NP), provide an interesting alternative to conventional vaccination approaches.

So far, nucleic acid-based vaccines have been delivered largely by intramuscular, intradermal, as well as subcutaneous injections, resulting in predominant transfection of myocytes [37] and keratinocytes [38], respectively. Whereas mRNA-based transgenes are expressed directly in the cytoplasm of the transfected cell [39], pDNA needs to translocate to the nucleus for transcription, followed by translation in the cytoplasm [40]. In case of intramuscular [41] as well as cutaneous [42] administration, directly transfected cells may express the antigen. Antigen may be transferred to regional APC by the release of exosomes [43] or apoptotic bodies [44]. In either case, antigen of exogenous origin is presented largely on MHCII, resulting in the activation of antigen-specific CD4^+^ T helper cells (Th) [45]. Only subpopulations of DC are characterized by so-called cross priming activity, which means that antigen is shuttled/processed in such a manner that MHCI is loaded, resulting in CD8^+^ T cell activation [46]. In case of direct APC transfection [47], the antigen is expressed and processed like an endogenous gene, resulting in parallel loading onto MHCI and MHCII molecules [48]. APC that are sufficiently stimulated by pathogen-derived molecular patterns (PAMP) or endogenous danger signals, mimicked by the adjuvant, upregulate expression of MHC molecules, of costimulators, and of soluble mediators (i.e., cytokines), and migrate into the secondary lymphoid organs (draining lymph nodes, spleen) to prime antigen-specific T cells [49]. Activated CD4^+^ T cells are required for full activation of CD8^+^ T cells to yield CTL [50], and to confer so-called B cell help [51]. Depending largely on the composition of cytokines released by activated APC, CD4^+^ T cells polarize into various types of Th [52]. In case of tumor responses, the induction of Th1 cells, depending largely on IL-12, is paramount for CTL activation [53].

### 2.1. Clinical Trials Using Nucleic Acid-Based Vaccines for Tumor Therapy

#### 2.1.1. pDNA Vaccines

In an early clinical phase I trial, stage IV melanoma patients were intranodally infused with pDNA encoding for melanoma-associated tyrosinase every two weeks for a total of four times [54]. This trial confirmed tolerability of pDNA administration, and some activation of tyrosinase-specific T cells, but no clinical responses were observed. In subsequent clinical trials DC were differentiated in vitro from peripheral blood monocytes of patients using GM-CSF (granulocyte macrophage colony-stimulating factor) plus interleukin (IL)-4, pulsed with tumor lysate/proteins, matured, and reinfused [55]. In order to evaluate the suitability of nucleic acid-based vaccination, in a clinical phase I/II trial that enrolled stage IV melanoma patients, monocyte-derived DC were transfected in vitro with pDNA encoding melanoma-associated antigens melan A and gp100 using a cationic peptide for pDNA transfer, and chloroquine to promote endosomal escape, and were stimulated with TNF (tumor necrosis factor)-α and IL-1β [56]. Patients were vaccinated every three weeks for a total of three months. Whereas antigen-specific T cell responses were observed, the clinical response rate was only in the range of 10%, and not sustained. So far, similar results have been obtained in most clinical studies on APC-focused pDNA vaccination (tabulated in [57]).

Only a few clinical trials have demonstrated therapeutic efficacy of pDNA vaccination. In a clinical phase I/II study, patients with carcinoembryonic antigen (CEA)-positive tumors (in most cases colorectal cancer) were repetitively treated with a pDNA vaccine that encoded for a MHCI-restricted CEA-derived peptide fused to an immunostimulatory domain derived from tetanus toxin fragment C as an adjuvant by intramuscular injection for three months [58]. About half of the patients developed diarrhea due to a break in tolerance towards CEA, which is also expressed by colonic mucosa. The group of patients that developed autoimmunity showed a prolonged overall survival over the 16 months observation period.

Several reports have shown that combined treatment with a pDNA vaccine and a second drug exerted improved anti-tumor responses. In a clinical phase I trial, progression of metastatic prostate cancer was attenuated in more than half of the patients vaccinated for three months with a prostate acid phosphatase encoding pDNA plus recombinant GM-CSF as an adjuvant, co-applied intradermally, in combination with the programmed cell death protein (PD-)1 blocking antibody pembrolizumab [59]. This effect was not observed in case of sequential treatment with the antigen encoding vector/GM-CSF for three months followed by pembrolizumab application. In a phase IIB/III trial, treatment of non-small-cell lung cancer patients with a vaccinia virus encoding the tumor-associated antigen (TAA) Mucin-1, and IL-2 to stimulate T cells (TG4010) by repetitive subcutaneous injections yielded longer overall survival of patients upon co-treatment with first line chemotherapy (different drugs) as compared to patients that received chemotherapy only [60]. The efficacy of TG4010 in combination with checkpoint inhibitors is evaluated in ongoing phase II trials (NCT02823990, NCT03353675).

Due to the overall low efficacy of pDNA vaccination in clinical trials observed so far, pDNA vaccines need to be improved to yield stronger immunogenicity. In the following various parameters that are important for the optimization of the design of pDNA vaccines as well as their delivery are discussed.

#### 2.1.2. mRNA Vaccines

Until a few years ago, mRNA-based anti-tumor vaccines were largely evaluated in clinical studies using patient-derived autologous DC electroporated in vitro with TAA-encoding mRNA either alone, in combination with adjuvant-encoding mRNA or followed by stimulation with soluble mediators, followed by intradermal administration. In most of these trials, adaptive antigen-specific immune responses were detectable, but only some reached clinical responses (the outcome of these clinical trials is listed in [61]).

In an early phase II clinical trial, acute myeloid leukemia patients were vaccinated by intradermal injection with autologous DC electroporated in vitro with mRNA, encoding Wilms´ tumor 1 (WT1) antigen in bi-weekly intervals for four cycles [62]. About a third of the patients displayed complete remission after more than a year after the first vaccination. Therapeutic efficacy of vaccination with WT1-mRNA transfected DC was increased by including the lysosomal targeting signal of lysosomal-associated membrane protein (LAMP) [63], which previously demonstrated to achieve improved loading of antigen onto MHCII [64]. Similar results were achieved in another clinical phase II trial on patients with acute myeloid leukemia (AML) using human telomerase reverse transcriptase (hTERT) encoding mRNA for ex vivo electroporation of DC, followed by intradermal application [65]. The hTERT expression unit was fused to a LAMP minigene. Transfected DC were applied weekly for six weeks, followed by bi-weekly application for another six rounds. Recurrence-free survival of accordingly treated patients was prolonged as compared to historical controls.

Therapeutic efficacy of ex vivo mRNA-vaccinated DC was also demonstrated for glioblastoma, applied after surgical removal of the major tumor mass, and in combination with more conventional treatment regimens. In a phase II clinical trial, autologous DC were electroporated ex vivo with mRNA derived from surgically removed glioblastoma, and were maturated with a cocktail of proinflammatory mediators prior to intradermal application [66]. DC were applied six weeks after surgery and combined radiotherapy/chemotherapy (temozolomide), twice in the first week, and once per month afterwards (up to 18 treatments). All patients received chemotherapy throughout the vaccination period. The group of DC-treated patients showed prolonged progression-free survival. Strongly improved progression-free survival of glioblastoma patients vaccinated in a similar setting was also observed in a phase I clinical study using an mRNA encoding cytomegalovirus (CMV) pp-65 for DC transfection and GM-CSF as an adjuvant [67]. CMV pp-65 was chosen based on the fact that glioblastoma cells expressed this protein, but no other brain cells [68].

In melanoma therapy, efficacy of mRNA vaccines was observed in a study that enrolled stage III/IV melanoma patients after resection of metastases [69]. Autologous DC were co-transfected ex vivo with a mixture of four to six melanoma-associated antigen-encoding mRNAs (MAGE-A1/A3/C2, Melan A, gp100, tyrosinase) plus a mixture of adjuvants (either the toll-like receptor (TLR) 3 ligand polyriboinosinic:polyribocytidylic acid (poly(I:C)) plus CD40 ligand-mRNA, or mRNA coding for CD40L, CD70, and a constitutively active TLR4 mutant (TriMix-mRNA)). Transfected DC were applied intradermally in a bi-weekly cycle up to 12 times, and interferon (IFN)-α 2b was administered concomitantly in most cases. Vaccinated patients showed an increased survival rate as compared to historical controls. In a follow-up study on stage III/IV melanoma patients, co-treatment of patients with DC co-electroporated with either of the melanoma antigen-mRNAs plus TriMix, and concomitant treatment with the checkpoint inhibitor ipilimumab (CTL-associated protein (CTLA-)4 blocking antibody), applied every three weeks for a total of four times yielded better long term survival rates than ipilimumab treatment alone [70].

Within the last few years also some clinical trials assessing the potency of systemically applied mRNA-based vaccines (e.g., NCT02410733; Lipo-MERIT) have been initiated, using lipoplexes to prevent mRNA degradation. The mRNA vaccine tested in the Lipo-MERIT study aims to directly target DC for melanoma therapy [71], and is comprised of several mRNAs that encode four different TAA (MAGE-A3, NY-ESO-1, TPTE, and tyrosinase,) to be presented via MHCI and MHCII, and induce IFN type I driven immune responses due to intrinsic stimulatory activity. In a preclinical setting, a liposomal formulation that specifically addressed DC was identified by testing the biodistribution and cell binding properties of a library of cationic liposomes consisting of DOTMA (1,2-di-O-octadecenyl-3-trimethylammonium propane) and DOPE (dioleoyl phosphatidylethanolamine), which differed in their size and zeta potential [72]. mRNA-loaded lipoplexes with a negative net charge and a diameter of around 300 nm almost exclusively accumulated in the spleen and were shown to address splenic and lymph node DC.

### 2.2. Optimization Strategies for Nucleic Acid-Based Vaccines

#### 2.2.1. Antigen

For tumor therapy, nucleic acid-based vaccines need to encode tumor-specific immunogenic peptides, which allows to comprise antigen-encoding sequences of different proteins within one minigene aimed to activate a broader number of CD4^+^ and CD8^+^ T cells [73]. In general, TAA may either constitute tumor-specific shared or tumor-specific unique antigens [74]. Whereas shared TAAs are also presented by normal cell types, albeit at lower extent, unique tumor antigens, also called neo-antigens, are exclusively expressed by tumors [75]. Especially in older studies, sequences encoding shared TAAs have been used [76], but this may also result in autoimmune responses [77]. On the contrary, effector T cell responses towards neo-antigens, identified in a tumor-specific manner by mutagenome analysis, have been reported to be more potent [78,79]. Moreover, antigens with a prolonged half-life have been shown to induce stronger CTL responses, and thereby increased immunogenicity [80]. To improve the presentation of (tumor) antigens, epitope-specific changes have been shown to increase MHC affinity [81], including the use of xenogeneic antigens [82]. Loading of pDNA-encoded antigens onto MHCII was also demonstrated to be improved by inclusion of the coding sequence of the invariant chain [83]. mRNA-encoded antigens were shown to be presented at higher extent via MHCII when fused with the lysosomal targeting signal of LAMP [64].

#### 2.2.2. Adjuvant

Conventional pDNA was reported to possess intrinsic immunostimulatory activity due to a CpG-rich motif located within the ampicillin-resistance gene that triggered TLR9 in endo/lysosomes [84]. Besides, pDNA was also shown to bind cytosolic DNA sensors that mediate activation of the stimulator of IFN genes (STING) signaling pathway [85]. Moreover, physical stress associated with vaccination may also exert adjuvant effects as observed for gene gun-mediated delivery of gold particle adsorbed pDNA into the skin [86].

However, nucleic acid-based vaccines normally contain an adjuvant, which is delivered as a separate unit, like the TLR3 agonist poly(I:C) [69] or CpG oligodeoxynucleotides (ODNs) that trigger TLR9 [87,88], or more conventional adjuvants like Alum [89]. Whereas these moieties trigger danger receptors, in several studies the efficacy of transgenes that encode constitutively active mutants of danger receptors like TLR4, and receptors with co-stimulatory activity like CD40L and CD70 [69] to confer APC activation has been evaluated. Additionally, minigenes encoding signaling adaptors and transcription factors have been assessed in this regard. For example, Shedlock and co-workers reported that co-transfection of an NF-κB p65 expression plasmid and of a HIV protein encoding pDNA by in vivo electroporation of mice yielded stronger T cell responses [90]. Likewise, human monocyte-derived DC co-transfected in vitro with an mRNA encoding for a constitutively active form of IKKβ (inhibitor of nuclear factor kappa B kinase subunit beta) showed elevated upregulation of surface activation markers and cytokines like IL-12, and conferred stronger activation of co-cultured CD8^+^ T cells [91] and NK cells [92]. Similarly, biolistic co-transfection of mice with an IRF-3 encoding pDNA enhanced T cell responses towards co-applied influenza antigen-encoding pDNA [93].

Further, the suitability of pDNA [49] or mRNA [94] encoding cytokines intended to activate APC and to modulate T cells (in a paracrine manner) has been evaluated. For example, Li and co-workers co-administered healthy volunteers a multigene HIV DNA vaccine plus an IL-12 encoding pDNA by intramuscular injection, which conferred increased Th1/CTL responses [95]. Bontkes et al. demonstrated that human DC co-transfected in vitro with a TAA, and IL-12 as well as IL-18 encoding mRNA induced increased activity of co-cultured CD8^+^ T cells and NK cells [94]. Similar findings were made in a preclinical mouse study upon intramuscular administration of a pDNA encoding mycobacteria antigen and IL-15, known to activate both APC as well as T cells and NK cells [96]. Further, administration of IL-2 and IL-7 pDNA expression constructs aimed to promote T cell activation and proliferation have been tested in preclinical studies [97].

#### 2.2.3. Inhibition of Regulatory Proteins in APC

**Figure 2:**
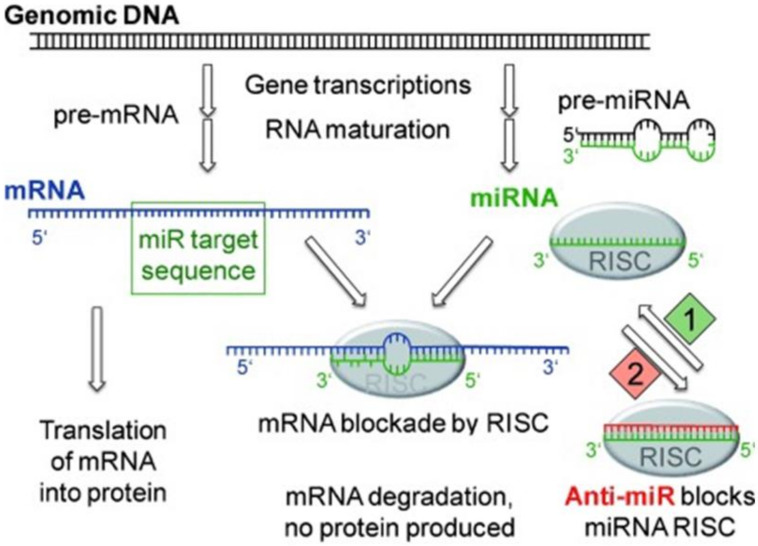
Mechanism of RNA interference (RNAi) and options for therapeutic intervention. (1) Substitution of tumor suppressor micro-RNA (miRNA, miR) in form of pre-miRNA or miRNA mimics, thereby inducing RNAi. (2) Blocking of oncogenic miRNA by miRNA-specific antagomirs (anti-miR). This figure is reprinted with permission from [103]. Copyright © 2020; John Wiley and Sons.

In other studies, the potential of small interfering RNA (siRNA) to inhibit the expression of endogenous inhibitory key factors in APC has been tested. For example, Luo and co-workers boosted anti-tumor responses using NPs that co-delivered the TLR3 ligand poly(I:C) and a siRNA specific for the transcription factor STAT (signal transducer and activators of transcription) 3 [98], which induces expression of anti-inflammatory factors like IL-10. Likewise, NPs delivering siRNA specific for the co-inhibitory receptor programmed cell death (PD) ligand 1 (PD-L1) have been evaluated in tumor studies [99]. More recently, also micro-RNA (miRNA) species, which constitute endogenously expressed small RNA species that inhibit gene expression (Figure 2), are considered interesting candidates to modulate the activation state of APC [100]. For example, delivery of a plasmid harboring multiple miRNA consensus bindings sites, termed miRNA sponge [101] and of anti-miRNA oligonucleotides [102], is intended to limit the inhibitory effect of miRNAs on activation-associated mRNA targets.

#### 2.2.4. Structural Optimization of pDNA Vaccines

##### Expression Units

In most of the aforementioned studies pDNA and mRNA species encoding antigen and adjuvant were applied as separate plasmids (in trans). However, the approach to integrate several transcription units into the same pDNA or mRNA in cis has received growing interest [104]. In case of pDNA, according vectors may either contain separate expression units each driven by another promoter, or a single promoter that regulates expression of the antigen and of molecular adjuvants. In case of the latter, which is also possible in case of mRNA vaccines, the different expression units may be separated either by an internal ribosomal entry site that confers cap-independent translation [105,106] or virus-derived recognition sites [107], which in the derived long peptide are recognized by a ubiquitously expressed protease [108].

##### Size Reduction

A large part of pDNA is of prokaryotic origin and is required only for propagation in bacteria. It has been shown that after transfection prokaryotic parts are silenced by formation of heterochromatin, which may spread into the eukaryotic expression unit(s), and thereby limit transgene expression [109]. Therefore, the strategy to flank the expression cassette comprised of the promoter and the transgene-encoding part with phage recombinase-recognition sites has received growing interest. This configuration allows deletion of the prokaryotic part in the late phase of plasmid propagation by inducing phage recombinase. In several studies the derived mini-circle DNA (mcDNA) was reported to yield a higher transfection efficiency as well as a longer duration of gene expression as compared to the full length parental construct [110].

##### Nuclear Transfer

Moreover, in case of a pDNA vaccine its nuclear translocation is necessary for transcription of the encoded transgene(s), which constitutes a hurdle in mitotically inactive APC [111]. It was shown that transcription factors may bind recognition sites within the gene regulatory regions of the pDNA, and mediate nuclear import of the pDNA by their nuclear localization signal (NLS) [112]. Especially the simian virus (SV)40 enhancer sequence was demonstrated to harbor several of these transcription factor binding sites, and inclusion of this region directly upstream or downstream of the transgene expression unit conferred enhanced nuclear import and elevated transfection efficiencies [40]. In a more controlled manner, viral peptides (e.g., SV40 large T antigen) coupled to pDNA can facilitate its nuclear entry via their NLS [113].

##### Transcriptional Regulation

Expression units of pDNA-based vaccines are often under transcriptional control of virus-derived promotors characterized by ubiquitous activity at high level, like the human intermediate/early CMV or the SV40 promoter [114]. Since viral promoters may be subjected to methylation-mediated inactivation, both eukaryotic promoters, like the human elongation factor (EF)1α or beta-actin gene promoter, as well as viral/eukaryotic hybrid (e.g., CMV/beta-actin) promoters have been introduced that allow long term transgene expression [115]. These types of promoters are still widely used in preclinical and clinical studies. On the contrary, the potential of promoters that restrict gene expression to DC to avoid unwanted vaccine expression by regulatory immune cells (e.g., TAM, MDSC) has been assessed in a limited number of preclinical studies only. The promoter of DC-STAMP (dendrocyte-expressed seven transmembrane proteins) is active in unstimulated human and mouse DC as well as in macrophages, and is downregulated upon stimulation [116]. Mice transduced with a lentivirus containing the DC-STAMP promoter displayed reporter activity in DC, monocytes, B cells, and NK cells [117]. Biolistic transfection of mice with a pDNA containing the promoter of the Langerhans cell (LC)-specifically active Dectin-2 gene resulted in LC-specific reporter activity [118], and when employed in a lentiviral vector conferred both DC- and macrophage-restricted reporter expression [119]. In several studies the promoters of the evolutionarily conserved human [120,121] and mouse [120,121] fascin-1 genes were demonstrated to restrict gene expression to maturing DC. Biolistic transfection of mice with fascin-1 promoter driven antigen encoding pDNA yielded largely DC-restricted transgene expression, and conferred Th1-polarized immune responses in models of allergy [122], and multiple sclerosis [123]. Furthermore, pDNA encoding for anti-inflammatory transforming growth factor (TGF)-β [123] and IDO [124] under transcriptional control of the fascin-1 promoter yielded tolerogenic effects.

#### 2.2.5. NPs for APC-Focused Delivery of Nucleic Acids

Biocompatible NPs are highly interesting for cellular transfer of nucleic acids [125] in the context of nucleic acid-based tumor therapy [126], since they offer protection against extracellular degradation by DNases [127] and RNases [128] either by dense complexation [129] or encapsulation [130] of nucleic acids. Especially in case of systemic application, NPs may confer either due to their intrinsic properties passive [72] or upon conjunction with surface receptor targeting moieties active [46] targeting of APC populations.

##### NP Size and Surface Characteristics Affecting Biodistribution

With regard to the design of NPs it needs to be taken into account that DC as the often preferred target cell type internalize smaller particles (<200 nm) more efficiently [131], whereas monocytes/macrophages preferably ingest larger ones (<5 µm) by means of receptor-mediated endocytosis and phagocytosis [132]. Besides size, also the shape of the NP may affect the efficacy of uptake as evaluated for gold-based NPs, which were engulfed by macrophages more efficiently in case of spherical as compared to e.g., elongated shape [133]. Both the cellular internalization of transfection complexes and the endosomal release of NP-complexed nucleic acids, can be increased by cell penetrating peptides (CPP) that are attached either e.g., to the pDNA [134] or to the NP [135].

Concerning the biodistribution of nucleic acid/NP transfection complexes, it was shown that small particles are easily transported into the lymph node, whereas larger particles remain longer at the site of administration [136]. Further, the route of administration can also account for the fate of NP delivery systems. After subcutaneous injection small PEGylated liposomes were found in a larger amount in the lymph node than after intravenous or intraperitoneal application [137]. Concerning NP clearance from the body, NPs that are smaller than 8 nm are cleared renally [138], and the extent of renal clearance was shown to correlate with the extent of negative charge [139]. Biliary clearance was observed especially for particles over 200 nm, and for strongly charged particles [140].

##### NP Types Suitable for APC Transfection

By now, a large variety of materials and structures has been evaluated for transfer of nucleic acids into APC, comprising inorganic materials like solid core gold [141] and iron oxide-based [142], mesoporous silica [143], and graphene oxide [144] based NPs. The latter have repetitively shown to confer endosomal escape of nucleic acids [145,146]. Polymer-based NPs bind nucleic acids by electrostatic interactions [147]. PLG (poly-D,L-lactide-co-glycolide) [148], PLGA (poly-D,L-lactic-co-glycolic acid) [149], and polyethylenimine (PEI) [150] are among the most intensely studied polymer-based NPs for delivery of nucleic acids. Of these, PEI by acting as a ’proton sponge’ conferred the most pronounced endosomal release of nucleic acids [151], but at the same time also mediated strong cytotoxicity [152]. Chitosan is a natural polysaccharide-based polymer, which has been evaluated for pDNA transfer [153] and similar to PLGA [154] was demonstrated to exert immunostimulatory activity [155]. Protein-based NPs offer the advantage of high biocompatibility [156]. For example, gelatin B (negatively charged) combined with protamine sulfate (positively charged) conferred DNA transfection, and mediated pDNA release under acidic conditions as apparent in endolysosomes [157]. Using endogenous proteins as nano-carriers may reduce potential immune reactions in response to repetitive application. In this regard, serum albumin coated with chitosan conferred DNA transfection [158]. NPs, consisting of albumin conjugated with cationic ethylenediamine complexed Bcl-2 specific siRNA, intravenously injected into mice with established melanoma lung metastases successfully inhibited further tumor progression [159]. Cationic lipids complex negatively charged nucleic acids by electrostatic interactions, and by interaction with the negatively charged cell membrane confer internalization of lipoplexes [160]. DOTAP (N-[1-(2,3-dioleoyloxy)propyl]-N,N,N-trimethylammonium chloride) was the first lipid to be used for pDNA transfection [161], and is still used either as a single component for complexation of nucleic acids or in combinations with helper lipids. With regard to the latter, neutral helper lipids like cholesterol have been included resulting in much stronger transfection efficiency presumably due to elevated endosomal escape of passenger DNA [162]. Incorporation of coiled-coil lipopeptides into liposomes resulted in direct release of the payload into the cytosol [163].

##### Administration Routes

Direct transfection of APC in secondary lymphoid organs can be achieved by intravenous application [164], given that the nano-vaccine predominantly addresses APC by passive [72] or active [165] targeting. This would result in the induction of antigen-specific T effector cells, which can home to each tissue, and thereby also reach metastases irrespective of their location. However, as delineated in preclinical rodent biodistribution studies, systemically administered NPs of larger size (≥ 200 nm diameter) may accumulate e.g., in lung as reported e.g., for mesoporous silica particles [166] or chitosan NP [167]. Moreover, most NP formulations tested so far accumulate in the liver [168] as a consequence of the general clearance function of the liver [169], conferred by Kupffer cells (KC) as the major liver-resident macrophage population [170] and liver sinusoidal endothelial cells (LSEC) [171]. KC and LSEC are equipped with a number of danger receptors, including different C-type lectin receptors (CLR) as e.g., the mannose receptor CD206 [172,173], and scavenger receptors that broadly bind negatively charged ligands [174,175]. Besides, KC [176] and LSEC [177] express high affinity Fc receptors, and KC also express complement receptors [178]. Therefore, it is conceivable that NPs, depending on the characteristics of the protein corona formed in vivo [179], also including complement activation as shown for lectin-coated NP [180], may be internalized preferentially by KC and LSEC. The formation of a pronounced protein corona may be attenuated by PEGylation, shown to reduce unwanted binding to KC [181] and LSEC [182], and by conjugation with CD47, which serves as a ‘do not-eat-me’ ignal for macrophages as evaluated for liposomes [183]. Furthermore, targeting moieties on NP may engage according receptors on either non-parenchymal liver cell population. An example is mannose, which has frequently been used to address CD206-expressing APC of myeloid origin [184,185].

NP delivery via the skin constitutes an interesting alternative to systemic NP delivery for several reasons: (i) topical application circumvents unwanted liver accumulation, (ii) cutaneous DC, comprising Langerhans cells (LC) as the epidermal DC population, which form a dense network (200–1000 LC/mm^2^ [186]), and dermal DC are apparent at rather high numbers in skin, and (iii) targeting is not necessary since only DC, at activated state, are able to migrate to secondary lymphoid organs [187]. By now, several approaches for transfection of skin DC have been tested successfully in clinical trials concerning safety and tolerability and are used in preclinical studies to evaluate vaccines. These include conventional intradermal injection [188], biolistic transfection of nucleic acids pre-adsorbed onto particles applied by gene gun [189] and PMED (particle-mediated epidermal delivery) [190], patches with dissolvable microneedles [191,192], and tattooing devices [193]. All of these transdermal delivery methods can transfer NP-complexed nucleic acids [194]. In case of biolistic transfection the method-associated physical stress was sufficient to confer activation and consequently emigration of transfected DC [86]. Further, administration of an electrical pulse just after intradermal [195] and intramuscular [196] injection, was shown to induce local inflammation, which activated APC at the according site, and to enhance overall transfection rates [197]. Consequently, electroporation devices that are applied in the context of intradermal injection are currently tested in clinical phase I (e.g., NCT04336410) and phase II (NCT03180684) pDNA vaccination studies.

Other potential delivery routes for tumor vaccination comprise the respiratory system by applying nebulized pDNA or mRNA that largely transfect lung epithelia [198], which has predominantly been employed for treatment of lung diseases like cystic fibrosis [199], and oral vaccination approaches using attenuated bacteria (e.g., *Salmonella typhimurium*) for pDNA transfer to APC in Payer’s patches [200].

##### Targeting of APC

Passive targeting of DC and monocytes/macrophages in vivo may be a consequence of the protein corona formed in case of many types of NPs due to adsorption of serum factors, which may constitute genuine ligands for cell surface receptors [179]. The composition of the protein corona is determined by several factors including e.g., charge and hydrophobicity of the particle surface. Further, serum factors due to interaction with the particle surface may alter their state of conformation, and thereby are recognized as ‘new’ ligands e.g., by scavenger receptors [201]. Finally, NPs may be recognized as pathogen-like by the innate immune system, e.g., in case of lectin surfaces intended to ensure biocompatibility of the NP, which however was found to trigger the lectin-dependent complement pathway [202]. This in turn resulted in adsorption of active complement C3 on the particle surface, and subsequent recognition of immune cells via complement receptors [180]. Unwanted adsorption of serum factors may be limited by conjugation with polyethylene glycol (PEG) [203]. However, concerning the repetitive application of vaccines potential adverse reactions as e.g., the induction of PEG-specific antibodies [204] need to be taken into account.

Active targeting of transfection complexes to DC and monocytes/macrophages can be achieved by conjugation of NPs with derivatives of natural ligands and antibodies that specifically bind endocytic surface receptors like C-type lectin receptors, which are expressed in a largely cell type-specific manner [205]. For example, the mannose receptor CD206 is highly expressed by macrophages (M2-like > M1-like), and is apparent at some extent on conventional DC [206], whereas DC-SIGN is predominantly expressed by conventional DC populations, but only by a low fraction of macrophages [207]. In a preclinical study, intramuscular vaccination of mice with mannosylated cationic liposomes (distearoylphosphoethanolamine-polycarboxybetaine/DOTAP/cholesterol) that showed intrinsic DC stimulatory activity and complexed a HIV antigen-encoding pDNA improved HIV-specific T cell responses [208]. More recently, trimannosylated liposomes (1,2-bis(hexadecyl)glycerol/1,2-Dioleoyl-sn-glycero-3-phosphocholine/cholesterol) were shown to specifically address DC-SIGN, and to accumulate at highest extent in the spleen after intravenous application, addressing predominantly DC [165]. While these approaches aimed to directly transfect APC in vivo, in an alternative approach Wang and co-workers designed a pDNA that encoded a fusion protein consisting of a tumor antigen polypeptide and a single chain antibody fragment known to bind the murine DC-specific receptor CD11c [209]. Thereby, this pDNA was aimed to be expressed in non-APC, but the expressed fusion protein was meant to target DC. In a mouse breast cancer model intramuscular injection of this pDNA prevented tumor growth when applied protectively prior to subcutaneous tumor cell inoculation, and attenuated tumor progression in a therapeutic setting. As mentioned above, lipoplexes composed of DOTMA and DOPE loaded with mRNA with a negative net charge and a size of around 300 nm due to these characteristics predominantly targeted DC in secondary lymphoid organs [72].

## 3. Inhibition of Regulatory Immune Cells

The success of vaccination to induce a sustained antigen-specific anti-tumor response is limited by regulatory immune cells that are induced and expanded by tumors as part of their evasion strategy [210]. Both MDSC [211] and Treg [212] can attenuate the T cell stimulatory activity of APC, the activation of T cells as well as the anti-tumor function of Teff, and effector functions of NK cells. To counteract the suppressive effect of regulatory immune cells the suitability of RNAi has been delineated [213,214]. Further, nucleic acids with immunostimulatory function were reported to reprogram MDSC to exert anti-tumor activity [215].

### 3.1. Inhibition of Treg by RNA Interference

Under homeostatic conditions Treg ensure tolerance towards self-antigens to prohibit autoimmunity [216], and against harmless antigens to prevent allergies [217]. Besides, as a negative feedback mechanism Treg are expanded and are also induced de novo in the course of immune reactions in order to limit immune responses and thereby to minimize tissue damages. Under healthy conditions Treg occur only in small numbers [218]. Depending on the place of origin Treg can be differentiated in thymic Treg (tTreg), alternatively termed natural Treg, [219] and in Treg that are induced in the periphery (iTreg) [220]. During thymopoiesis thymocytes, which express a T cell receptor (TCR) with intermediate affinity for self-peptides, differentiate into immunosuppressive tTreg [221]. iTreg derive from CD4^+^/CD8^+^ T cells, whose TCR is not specific for self-antigens, but recognizes microbiota- and environmental antigens presented by DC in the periphery [222] in the context of low co-stimulation and/or Treg-promoting factors like retinoic acid, kynurenine, and TGF-β [223,224]. In mice, tTreg and some iTreg populations can be identified by constitutive expression of the IL-2 receptor CD25 and by co-expression of the transcription factor FoxP3 [225], whereas other iTreg populations are Foxp3-deficient, but may express anti-inflammatory mediators like IL-10 and TGF-β [226].

In cancer, constituents of the TME produce anti-inflammatory mediators, which promote Treg expansion/induction in the periphery [218], and release of chemokines as for example the C-C motif chemokine ligand (CCL) 22, to recruit Treg to the tumor [227]. Treg suppress anti-tumor responses on the level of APC activity, T cell activation, T effector cell functions, and the functions of NK cells by numerous mechanisms, as for example anti-inflammatory cytokines (e.g., IL-10, TGF-β), surface receptor interactions (e.g., negative cross-talk via CTLA-4), IL-2 depletion, and transfer of cyclic adenosine monophosphate (cAMP) [228].

There are different approaches to overcome the obstacle of Treg-mediated suppression of anti-tumor responses, including strategies to deplete Treg or to reduce their suppressive activity [229]. Concerning nucleic acid-based approaches to attenuate Treg induction, silencing of tumor-derived TGF-β in murine CT26 colon carcinoma cells by transfection with oligofectamine/TGF-β1 siRNA complexes suppressed Treg induction [230]. Most recently, Masjedi et al. reported that ex vivo silencing of the adenosine A2A receptor (A2AR) with an A2AR-specific siRNA complexed with PEG-chitosan-lactate (PCL) NPs inhibited the differentiation of CD4^+^CD25^−^ T cells derived from 4T1 breast tumor-bearing Balb/C mice toward Treg [231]. Alternative approaches have aimed to minimize the suppressive capability of Treg. For example, in vitro transfection of murine Treg with a Foxp3-specific siRNA resulted in profound inhibition of their suppressive capacity [232]. Another treatment option is to interfere with the recruitment of Treg to the tumor site. Kang and co-workers have demonstrated that tumor infiltration with Treg in athymic nude mice, inoculated with human breast cancer cells, can be prohibited by tail vein injection of Treg transfected with a siRNA specific for CCL22 [233]. Besides the use of synthetic siRNA for RNA interference, in recent years miRNA (over)expression intended to alter the genetic program of Treg has gained increasing interest. In this regard, lentiviral transduction of Treg in vitro with miR-9 and miR-155 encoding vectors resulted in reduced expression of CTLA-4, which is a key factor for the immunosuppressive activity of Treg [234]. Additionally, Jonuleit et al. reported that in a mouse melanoma model systemic delivery of CTLA-4 specific siRNA by cationic lipid-assisted PEG–poly(lactic acid (PLA))-based NP resulted in reduced Treg numbers, and inhibited tumor growth [235]. Administration of miR-141 and miR-200a mimics in multiple sclerosis patients shifted the differentiation of naive T cells towards Th17, and at the same time inhibited Treg differentiation [236]. In a mouse model of epithelial ovarian cancer (EOC) in vitro transfection of CD4^+^ T cells with miRNA 29a-3p and miR-21-5p mimics, complexed with the commercially available X-tremeGENE siRNA transfection reagent, followed by adoptive transfer into tumor-burdened mice, tumor growth was attenuated [237]. This outcome was based on the inhibitory effects of both miRNA species on STAT3 expression, thereby favoring Th17 over Treg differentiation. In another study, transfection of Treg with miR-142-3p reduced the level of intracellular cAMP and adenylyl cyclase type 9 expression, which impaired their suppressive properties [238]. Treg-specific delivery of biologicals may be achieved by using IL-2-functionalized NPs as shown for hydroxyethyl starch nanocapsules that targeted Treg due to their constitutive high level expression of the IL-2 receptor CD25 [239].

Altogether, these studies demonstrate that nucleic acid-based strategies have a high potential to reduce overall Treg activity in cancer. However, it should be noted that Treg depletion may result in a compensatory induction of MDSC [240].

### 3.2. Strategies for MDSC Reprograming and Depletion

MDSC derive from myeloid precursor cells during myelopoiesis [241]. Immunomodulatory factors generated by tumors like some cytokines, chemokines, or colony-stimulating factors (CSF) are capable of stimulating expansion of MDSC on the expansion of monocytes, conventional DC, and neutrophils [242], while chronic inflammations can lead to extramedullary myelopoiesis [243]. The expansion and activation of generated MDSC requires concerted interaction of several signaling pathways, like the NF-κB, JAK-STAT, HIF-1α, C/EBPβ, and CHOP pathway. Based on the expression of plasma membrane markers, the amount of immune suppressive molecules as well as by functional analysis [244], MDSC can be allocated to CD11b^+^Ly6G^−^Ly6C^hi^ monocytic (m)MDSC and to CD11b^+^Ly6G^+^Ly6C^low^ granulocytic (g)MDSC [245]. MDSC exert potent immune-suppressive activity against T cells [246] and NK cells [247]. Accordingly, MDSC contribute to control autoimmunity [248] and infections [249]. After activation, MDSC migrate to the site of inflammation or to the tumor site in response to a variety of chemokines [250]. There, MDSC generate an immune-suppressive milieu, which is enhanced by different cytokines [243]. The infiltration of mMDSC into a tumor leads to a distribution of tumor cells from the place of origin by induction of epithelial–mesenchymal transition (EMT), which generates a cancer stem cell (CSC) phenotype [251]. Tumor infiltration of gMDSC withdraws the CSC phenotype and leads to tumor cell proliferation and promotes metastasis. In secondary lymphoid organs MDSC suppress APC, the activation of tumor antigen-specific T cells, and T effector cells by several mechanisms in an analogous manner as described for Treg [252].

In some approaches siRNA and miRNA have been applied to attenuate MDSC generation and their suppressive activity. For example, Boldin et al. have shown that miR-146a inhibited the proliferation of MDSC by targeting tumor necrosis factor receptor-associated factor 6 (TRAF6) and IL-1 receptor-associated kinase 1 (IRAK1) [253]. Similarly, miR-424 was reported to interfere with MDSC differentiation [214]. In several mouse tumor models intravenous application of oligofectamine/miR-223 complexes inhibited tumor-conferred MDSC generation by targeting myocyte enhancer factor 2C (MEF2C) in bone marrow progenitor cells [254].

Moreover, some types of NPs as an intrinsic property have been reported to reprogram MDSC towards proinflammatory macrophages as shown e.g., for cationic dextran- and PEI-based NP [215] in vitro and for NP modified with a cationic polymer in vivo [255]. In addition, TLR agonists that address TLR7/8 (e.g., R848) and TLR9 (CpG ODN) were shown to exert similar effects both in vitro and in vivo [256], which may contribute to the overall immunostimulatory effect of these adjuvants.

### 3.3. Inhibition of Treg and MDSC by Tumor-Directed Approaches

Besides direct targeting of Treg and MDSC via RNA interference, the induction/expansion and tumor infiltration of either regulatory cell type may also be controlled indirectly by affecting tumor gene expression and as a secondary effect in the course of inducing anti-tumor responses. Stem cell factor (SCF; c-kit ligand) is generated by tumors and confers MDSC infiltration [257]. In a mouse MCA26 colon tumor model adenoviral transfer of SCF-specific siRNA resulted in reduced accumulation of MDSC at the tumor site [258]. Injection of a TNFAIP3-specific siRNA into E.G7 or B16-F10 melanoma induced apoptosis in MDSC via activation of the c-Jun N-terminal kinase (JNK) pathway [259]. Injection of vascular endothelial growth factor (VEGF)-specific siRNA, complexed with nanogels, into renal tumors significantly reduced MDSC numbers in that area [260]. Injection of a Newcastle Disease Virus Hemagglutinin–Neuraminidase encoding pDNA into the ear pinna of DA3 tumor bearing Balb/c mice promoted innate anti-tumor responses and reduced MDSC infiltration into the tumor site [261]. In humans suffering from pancreatic ductal adenocarcinoma (PDA), in many cases antibodies and T cells specific for α-enolase (ENO1) have been identified [262]. In a mouse model of autochthonous pancreatic cancer, injection/electroporation with a ENO1-encoding pDNA attenuated tumor growth and concomitantly also the expansion of Treg and MDSC [263].

## 4. Generation of T Cells and NK Cells Expressing CARs for Tumor Therapy

CARs are synthetic antigen receptors, which comprise an extracellular antibody domain, a transmembrane domain, and an intracellular signaling domain, and recognize e.g., tumor-associated antigens [264]. So-called CAR T cells (CAR-T) and CAR natural killer cells (CAR-NK) are generated by transfection of either cell type with a CAR-encoding pDNA, mRNA, or are transduced with a CAR-encoding viral vector [265]. Therefore, CAR expressing cells are able to recognize antigens under tumor-induced immune-suppressive conditions and can exert a proper immune response. For CAR synthesis, the variable domains of an antibodies’ light and heavy chain are fused, for example by short glycine-serine linkers, to yield a single chain fragment variable (scFv) [266]. The transmembrane domain is usually derived from CD28 or another membrane receptor [267]. In most cases CD3ζ, which is a component of the endogenous TCR, serves as the signaling domain for CAR-T [268]. For CAR-NK the transmembrane immune signaling adaptor chain is employed as the signaling domain [264]. The signaling domain is often combined with one or more co-stimulatory motifs [269] like CD28 [270], CD137, CD357, CD278, or CD134 [271] for CAR-T, and CD28, CD137 [272], CD278, CD134 [273], or Dap10 [270] in case of CAR-NK. The first generation of CARs contained only CD3 (ζ or γ chain) signaling motifs, which are able to activate murine CTL hybridoma cells, modified with chimeric genes for surface receptors, e.g., to trigger IL-2 secretion, but these may be inactivated by tumors [274]. The second generation of CARs was equipped in addition with a co-stimulatory domain, and the third generation possessed more than one co-stimulatory domain [275].

In an alternative approach, the signaling and the co-stimulatory domains are split between two different CARs, which is termed combinatorial targeting [264]. Until now, two CAR-T based immunotherapies have been approved by the United States Food and Drug Administration (FDA). Both are CD19-directed CAR-T immunotherapies, targeting the pan-B cell receptor CD19. They have shown significant results in the treatment of non-Hodgkin lymphoma (NHL), acute lymphoblastic leukemia (ALL), and chronic lymphocytic leukemia (CLL) [276]. Of these, treatment with tisagenlecleucel (T cells from the patients’ blood are lentivirally transduced with CD19-speciic CARs) yielded an overall remission rate of 81% after three months in patients suffering from relapsed or refractory ALL, but caused serious, mainly reversible toxic effects in children and young adults under 25 years [277]. In patients with NHL, axicabtagene ciloleucel (lentiviral transduction of patients’ blood T cells with CD19-specific CARs) resulted in an objective response rate of 82%, and a complete response in 54% of cases [278]. However, treatment with either CAR-T treatment can lead to serious and even life-threatening side effects, like the tumor lysis syndrome, a disease which can result from a tumor therapy, causing hyperuricemia, hyperkalemia, hyperphosphatemia, and hypocalcemia [279], and the cytokine release syndrome that is induced by a cytokine storm [280], leading to fever, hypotension, and respiratory insufficiency [281].

Another problem of CAR-Ts is the interaction of MDSC with CAR-Ts, which may lead to a reduction of CAR-T activation, to reduced proliferation after antigen stimulation, and lowered cytokine production [282]. MDSC in the liver for example suppress an anti-tumor response of CAR-Ts via binding of PD-L1 that engages PD-1 on T cells [283]. The expression of PD-L1 by MDSC in the liver is supported by GM-CSF and is largely regulated by the transcription factor STAT3. The negative effect of MDSC on CAR-T can be avoided by MDSC depletion, using therapeutic drugs like gemcitabine and 5-fluorouracil [284], neutralization of GM-CSF, e.g., by otilimab that is currently assessed in a clinical phase 3 study [285], and PD-L1 blockade, e.g., by checkpoint inhibitors like atezolizumab [286]. For example, Fultang and co-workers have recently shown that the activity of an anti-GD2-/mesothelin-/EGFRvIII-CAR-T was significantly enhanced when co-applied with the anti-MDSC drug gemtuzumab ozogamicin, an anti-CD33 antibody linked to cytostatic calicheamicin [287]. Altogether, due to the high potential of CARs for cancer treatment, improvement of CAR-based therapy is in the focus of research. For example, Wang et al. have recently generated CAR-T cells by electroporation-based transfection of T cells with non-viral mcDNA, which is considered much safer than virus-based chimeric antigen receptor-engineered CARs [288].

## 5. Manipulating the TME Using Therapeutic Nucleic Acids

The TME is a complex, very heterogeneous network of stromal and endothelial cells as well as recruited immune cells [289]. It is characterized by leaky blood vessels, a special tumor-specific extracellular matrix (ECM), immunomodulatory agents/cytokines, and growth factors [18,289,290,291]. The TME plays an important role during tumorigenesis as well as tumor progression and metastasis by supporting the tumor cells in evading the immune system [19,292] and by contributing to chemoresistance [293]. Different cell types like CAF [294], TAM (pro-tumoral phenotype) [295,296,297], MDSC, and Treg (see Section 3) [18] as well as tolerogenic DC [18,298] contribute to the establishment and maintenance of the immunosuppressive tumor surroundings. In addition, the TME inactivates effector functions of tumor-infiltrating lymphocytes (TIL) by various mechanisms, and thus undermines immunosurveillance [292,299,300].

Further characteristics of the tumor tissue comprise acidity (≈pH 6.5) due to the Warburg effect [301,302], hypoxia [303], expression of distinct matrix enzymes like matrix metalloproteases (MMPs) [304], and an elevated redox potential [305] as well as increased levels of reactive oxygen species (ROS) [306]. These properties display barriers in the delivery process of anti-tumor drugs, but can be also exploited for bio-responsive targeting of therapeutics to the tumor tissue [307]. By this, tumor selectivity and overall biocompatibility might be enhanced. Besides, passive targeting via the enhanced permeation and retention (EPR) effect [308], and more effective tumor addressing via tumor homing peptides and CPPs can increase accumulation of (nano) formulations within the tumor [309,310,311]. In addition, active targeting mediated by ligands such as peptides, vitamins, or antibodies is often utilized to direct a therapeutic selectively to the target site [312].

Immunotherapeutic approaches often aim to evoke a switch from immunosuppression to immune permission within the tumor tissue. By this, the tumor becomes immune-sensitive again, and then can be effectively combated by the innate and adaptive immune system. In the following, a selection of diverse strategies for TME manipulation is presented with a focus on nucleic acid-based approaches.

### 5.1. Modulation of Intratumoral Signaling by Nucleic Acids

In the immunosuppressive TME a disproportion exists between soluble mediators (cytokines and growth factors) exerting pro- and anti-inflammatory properties, thereby promoting tumor immune escape and tumorigenesis [313]. There are two options to counteract this imbalance, resulting in effective anti-tumor activity [313]. On the one hand, the immune system can be stimulated by overexpression of pro-inflammatory cytokines. On the other hand, immunosuppression can be reduced by inhibition/neutralization of anti-inflammatory signals.

Cytokines are key mediators in the communication of immune cells and are crucially involved in controlling the intensity of an immune response [314,315,316]. Thus, it is not surprising that cytokine therapy has been pursued as a cancer immunotherapeutic approach for more than 30 years now. However, in clinical studies, such cytokine therapies have not met the expectations based on the results of preclinical studies, especially when applied as monotherapies [313]. Only IFN-α [317] and IL-2 as high-dose therapy [318] have been approved for the systemic treatment of several cancers, based on moderate beneficial anti-tumor effects in clinical trials. Ongoing research is focused on increasing therapeutic efficacy and biocompatibility by developing recombinant cytokines with improved pharmacokinetics (e.g., PEGylated or fused with targeting antibodies), combinations with other immunotherapeutic approaches such as immune checkpoint inhibitors, and local or specifically targeted administration of (recombinant) cytokines [313]. Besides that, cytokine gene therapy (using gene encoding pDNA or viral vectors) and other nucleic acid-based approaches (like RNAi or genome editing) are promising concepts [24,313,316]. Table 1 summarizes such nucleic acid-based approaches evaluated in clinical trials. In the following, important signaling molecules and strategies (especially therapeutic nucleic acids) to modulate their levels within the tumor tissue are outlined.

IL-2 stimulates T-lymphocytes and NK cells, but also controls the duration and intensity of their activation, regulates immune homeostasis, and balances the Teff/Treg ratio [313,334]. Various autologous/syngeneic as well as allogeneic IL-2 gene-modified tumor cell vaccines have been investigated in preclinical and clinical studies for their potential in prophylactic and therapeutic application for the treatment of advanced and metastatic cancers like melanoma [319,320,321,322,335,336,337]. In vitro transduction of tumor cells with the IL-2 gene was achieved using viral vectors (e.g., retro-viral or adenoviral) [320,322,336] or by employment of advanced methods like the adenovirus-enhanced transferrinfection (AVET) system [319,335,337,338]. The toxicity profile of systemic IL-2 gene therapy can be improved by transcriptional targeting of IL-2 to the tumor to ensure specific expression of the IL-2 gene within the tumor [339,340,341].

TNF-α exhibits tumoricidal effects by inducing apoptosis and hemorrhagic necrosis of tumor cells [323]. GenVec’s TNFerade is a replication-deficient adenoviral vector encoding for TNF-α under the control of a radiation-inducible promotor [323,324], applied by intratumoral injection. Phase III clinical trials have been terminated in 2010, as a study in locally advanced pancreatic cancer failed to show a significantly improved outcome of combination therapy with TNFerade in comparison to standard therapy alone [342]. Reduced transgene expression may be caused by (pre-existing) immune responses against the adenoviral vector, mainly mediated by antibodies, limiting the option of repeated application [343]. Besides viral vectors, non-viral carrier systems for TNF-α delivery are subject of research as well. Kircheis et al. for example designed surface-shielded transferrin-PEI/DNA complexes for targeted TNF-α gene delivery after intravenous application in tumor-bearing mice [344]. Significant and selective TNF-α expression within the tumor without detectable serum levels could be demonstrated in three different tumor models. In a combination approach, Su et al. evaluated to which extent systemic TNF-α gene therapy synergized with liposomal doxorubicin (Doxil^®^) to enhance tumor endothelium permeability, and thus would promote accumulation of the chemotherapeutic drug within the tumor [345]. Synthetic polymers based on amino ethylene units [346,347] were used as pDNA carriers. The beneficial effect of TNF-α expression on concomitant Doxil^®^ therapy was proven in all tested tumor models including metastases [345]. All in all, this combination approach offers great potential in treating metastases even with low doses of chemotherapeutic drugs. Quinn et al. achieved synergistic effects on tumor growth inhibition by combining systemic application of a previously evaluated RGD-targeted adeno-associated virus phage encoding for TNF-α [348,349] with hypo-fractionated radiation for the therapy of disseminated melanoma [350].

Another interesting candidate for cancer immunotherapy is IL-12 because of its ability to activate both the innate and the adaptive immune system [351]. In addition, IL-12 has anti-angiogenic properties by inducing IFN-γ, which in turn inhibits VEGF and MMPs [313,351,352,353,354]. In early clinical trials, however, the anti-tumor activity of systemically applied IL-12 was found to be only moderate, and was accompanied by severe side effects [351,355]. IFN-γ as induced by IL-12 is mainly responsible for the dose-related and schedule-dependent toxicity [353,356]. New strategies focus on targeted and local delivery of IL-12 to minimize systemic toxicity and to improve specific tumor targeting by conjugating IL-12 to tumor antigen-specific monoclonal antibodies (so-called immunocytokines) [357,358]. Moreover, various IL-12 gene therapy approaches ex vivo and in vivo are pursued [359,360]. Different delivery methods comprise viral vectors like adeno- or retroviral vectors [325,361,362,363,364,365,366], and non-viral techniques such as electroporation [367,368,369,370,371,372,373] or synthetic carrier systems like (lipo)polymer-DNA complexes and liposomes [374,375,376,377]. In order to increase the specificity of local IL-12 expression within the tumor, an IL-12 transgene with a ligand-inducible expression switch was designed [325,364]. Another way to locally control in situ expression of IL-12 is to engineer CAR- T cells, which release IL-12 in an inducible or constitutive manner [378]. Moreover, the IL-12 gene may be inserted in the genome of oncolytic viruses as an immune stimulatory component (see Section 5.3).

GM-CSF has been investigated as an adjuvant for different types of vaccines because of its stimulatory effect on myeloid cell types like conventional DC and macrophages [313]. Unfortunately, GM-CSF activates TAM and MDSC as well, thereby supporting tumor growth. These opposing effects are mainly responsible for its only moderate clinical efficacy [316]. Combination therapy is an option to overcome this issue; e.g., co-treatment with recombinant GM-CSF and immune checkpoint inhibitors led to prolonged survival of metastatic melanoma patients [379]. An example for a GM-CSF gene-based approach is the GVAX technology [326,327,328,329]. To this end, allogeneic pancreatic tumor cells have been transfected ex vivo with pDNA encoding GM-CSF. GVAX has been tested in combination with immune checkpoint inhibitors as well as with tumor vaccines. Moreover, oncolytic viruses often encode inter alia for GM-CSF (see Section 5.3). GM-CSF is also addressed in strategies to improve the efficacy and to lower the toxicity of CAR-T cell therapies. However, in contrast to the aforementioned GM-CSF therapy concepts, here GM-CSF is not substituted, but knocked out for example via CRISPR/Cas9 technology [380].

The CXCL12/CXCR4 (C-X-C motif chemokine 12/C-X-C motif chemokine receptor 4) axis plays a crucial role in tumorigenesis, metastasis, and chemoresistance [381,382], and therefore is an ideal target for cancer immunotherapy. However, the toxicity of systemic anti-CXCL12 therapy approaches using small CXCR4 inhibitors like AMD3100 [383] and monoclonal antibodies targeting CXCL12 [384] is a serious issue. Transient and locally restricted expression of antibody-like trap proteins that bind and neutralize CXCL12 constitutes an option to increase systemic tolerability [385,386]. For this purpose, NPs are used for target site-selective delivery of pCXCL12-trap encoding pDNA [18], such as lipid NPs/liposomes [385,386,387].

VEGF is crucial for neoangiogenesis, which is essential for tumor progression and metastasis [388]. Moreover, VEGF contributes to immunosuppression within the TME [389]. Accordingly, several anti-VEGF therapeutics have already been clinically approved, and many pre-/clinical trials are currently carried out evaluating the VEGF trap protein aflibercept [390,391] or monoclonal antibodies that target either VEGF itself (bevacizumab) [392,393] and its receptor VEGFR (ramucirumab) [394] in combination with classical chemotherapeutics or immune checkpoint inhibitors [389,395]. Another potent strategy is RNAi aimed to knock-down VEGF or VEGFR, which showed good anti-tumor results in many preclinical studies. In this regard, CPPs [396,397,398,399], polymers like PEI [400,401,402,403] or chitosan [404], cationic liposomes [405,406], gold [407], and graphene oxide NPs [408], often modified with shielding and targeting units, have been used as delivery systems.

TGF-β exhibits manifold functions regarding cell proliferation, differentiation, migration, and apoptosis [409,410,411]. In the context of cancer progression, an overexpression of TGF-β has been observed within the TME, promoting EMT, immunosuppression, and metastasis. However, these tumor-promoting effects of TGF-β occur only in late-stage tumors, while in early stages its anti-tumor activity is more pronounced. Thus, anti-TGF-β therapy approaches aim to treat advanced cancers. A lot of preclinical and clinical research has been performed in the field of nucleic acid-based strategies ranging from siRNA [412] over miRNA [413,414,415] to antisense oligonucleotides (ASO) [416,417,418,419,420,421]. Belagenpneumatucel-L is an anti-TGF-β allogeneic tumor cell vaccine, based on non-small cell lung cancer cells genetically engineered to express ASO directed against TGF-β [331,332,333]. In a phase III clinical trial, however, no significant increase in the mean overall survival was achieved compared to placebo treatment, but e.g., prior treatment with radiation therapy was found to have a positive effect on therapeutic outcome [333]. Therefore, further investigation in clinical trials is necessary.

In addition to the mediators discussed above, many others can be addressed in immunotherapeutic approaches as well [313,316]. For example, intramuscular IL-27 and intratumoral IFN-α gene delivery via viral vectors promoted Treg depletion in the TME [422,423,424]. This is favorable in view of the efficacy of cancer immunotherapy [425,426], suggesting that both approaches are valuable as adjuvant therapies. Moreover, IFN-α showed strong anti-proliferative, anti-angiogenic, and immunomodulatory activity [427,428]. An IFN-α encoding adenoviral vector (rAdIFNα2b/Syn3, Instiladrin^®^) has been investigated in advanced clinical trials for intravesical treatment of BCG (Bacillus Calmette–Guerin) unresponsive bladder cancer [330]. Results of a phase III clinical trial that has been completed in 2018 are still pending (NCT02773849).

### 5.2. Nucleic Acid-Mediated Immune Checkpoint Inhibition and T Cell Stimulation

Immune checkpoints regulate the intensity and the duration of immune responses [429,430]. By this, self-tolerance is preserved, and hence tissue damage is minimized. Tumors often abuse such pathways in order to create an immunosuppressive surrounding, e.g., by anergizing tumor-reactive Teff. Consequently, blockade of immune checkpoints presents a very promising method to restore immunity against the tumor and the TME. Among these CTLA-4 and PD-1 are the best characterized receptors [431,432]. Intensive research led to therapy concepts of immune checkpoint inhibition, which revolutionized treatment especially of advanced cancers [433]. Up to now, several antibodies addressing CTLA-4, PD-1, and its ligand PD-L1 have been implemented in cancer therapy regimens [25,26]. However, response rates are quite low, and relapse often occurs due to resistance development [434,435,436,437,438]. Moreover, immune checkpoint blockade is effective only if the number of tumor-reactive Teff is high enough at the beginning of treatment [434,439,440,441,442]. In this regard, the T cell number in a patient can be increased by ex vivo expansion of TIL that are subsequently reinfused, or by prior treatment with tumor vaccines [438]. Combinations of different immune checkpoint inhibitors as well as their combination with other (immuno)therapeutic approaches aim to overcome the resistance mechanisms [436,437,442].

**Figure 3:**
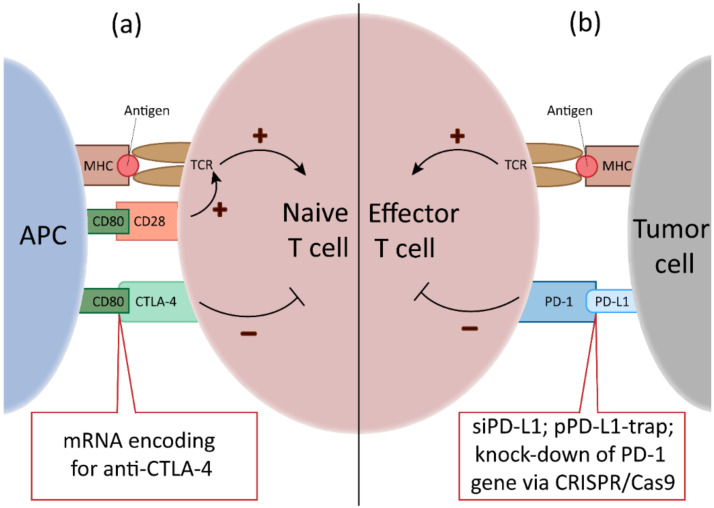
Immune checkpoint inhibition mediated by nucleic acid-based strategies. (**a**) Besides recognition of major histocompatibility complex (MHC)-bound antigen on the surface of APC via TCR, co-stimulatory signals—inter alia interaction of CD80 (B7-1) and CD28—are required for full T cell activation. The duration and intensity of activation is regulated among other things by immune checkpoint CTLA-4 that binds with high affinity to CD80. Blocking of this interaction results in enhanced T cell activity. One therapeutic option is delivery of mRNA encoding for anti-CTLA-4 antibodies. (**b**) Tumor cells often upregulate PD-L1 that binds to PD-1 on effector T cells, thereby inhibiting the activity of effector T cells. Nucleic acid-based approaches for blocking this immune checkpoint comprise siRNA against PD-L1, pDNA encoding for PD-L1 trap proteins (pPD-L1-trap), and CRISPR/Cas9-mediated knock-down of PD-1 gene.

Other major issues of checkpoint inhibitor therapy are immune-related adverse effects and toxicity [443,444]. Systemic toxicity can be reduced by targeted delivery of checkpoint inhibitors using NPs and by nucleic acid-based approaches [442]. Concerning the latter, mRNA encoding for an anti-CTLA-4 antibody [445], pDNA encoding for PD-L1 traps [386,446,447], siRNA specific for PD-L1 [448,449,450,451,452], and CRISPR/Cas9-mediated knock-out of the PD-1 gene in CAR-T cells [453,454] have been tested so far (Figure 3).

For example, Pruitt et al. electroporated DC ex vivo with mRNA encoding heavy and light chains of blocking antibodies specific for CTLA-4 and glucocorticoid-induced TNFR-related protein [445], which are expressed by Treg at high level [455]. Transfected DC were co-administrated with tumor antigen-transfected DC via subcutaneous injection into B16/F10.9 melanoma bearing C57BL/6 mice [445]. Based on the encouraging results, a phase I clinical trial for treatment of metastatic melanoma has been initiated (NCT01216436).

Transient local expression of PD-L1 trap was pursued by Huang and co-workers [447]. For this purpose, pDNA encoding for PD-L1 trap fusion protein was loaded into lipid-protamine-DNA NPs, consisting of a DNA-protamine core within pre-formed DOTAP-cholesterol liposomes. These were optionally equipped with 1,2-distearoylphosphatidylethanolamine (DSPE)-PEG or DSPE-PEG-AEAA for shielding and targeting. These nano formulations were applied intravenously in combination with intraperitoneally administered oxaliplatin, a chemotherapeutic drug inducing immunogenic cell death and thereby activating DC. By this approach, synergistic effects on tumor inhibition were achieved in a colorectal cancer mouse model.

A further combination approach was conducted by Zhou et al. by combined administration of doxorubicin and of PD-L1-specific siRNA delivered by stimuli-responsive NPs in a B16 melanoma tumor model [452]. These NPs were dually sensitive towards the extracellular slightly acidic pH of tumor cells (pH-triggered detachment of the PEG layer) and their elevated intracellular redox potential (reduction-sensitive polymer core of poly-L-lysine–lipoic acid). This combination therapy was superior to either monotherapy in terms of specificity, efficacy, and tolerability, proving once more the advantage of targeted combination therapies.

### 5.3. Multi-Faceted Combat of Cancer by Oncolytic Virotherapy

Oncolytic viruses may constitute the next breakthrough in cancer immunotherapy [456]. They comprise DNA and RNA viruses, which can be wild-type (e.g., coxsackie virus, reovirus) or genetically modified (e.g., herpes simplex virus (HSV), adenovirus, vaccinia virus) [457]. Oncolytic viruses selectively replicate in tumor tissue while destroying it [14,458,459,460]. Moreover, they exhibit an immunostimulatory function. Infection and lysis of tumor cells lead to the release of ROS and proinflammatory cytokines as well as danger-associated molecular patterns and intracellular tumor antigens, stimulating both the innate and the adaptive immune system [460]. By this, even immunological memory can be induced, resulting in long-lasting anti-tumor effects [14,461].

**Figure 4:**
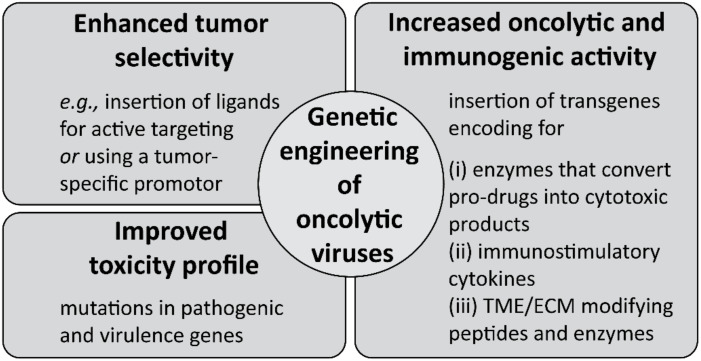
Genetic modifications to enhance selectivity, safety, and efficacy of oncolytic virotherapies.

In 1991, Martuza et al. succeeded in producing the first genetically modified HSV-1 characterized by a mutation in the thymidine kinase (TK) gene to ensure selective replication only in tumor cells [462]. This pioneer work opened a new way for cancer treatment. The first clinical trial with an oncolytic virus started in mid-1990 [463], followed quickly by many others [464]. However, the clinical efficacy fell short of the expectations, but safety and synergism with standard cancer treatments could be demonstrated [464]. Subsequent generations of oncolytic viruses have been developed by genetic engineering to enhance selectivity and efficiency while maintaining or even improving safety [457,459,465,466] (Figure 4). Tumor selectivity can be enhanced at several levels (transduction, transcription, translation, post-translation) as well as via oncogenic targeting or insertion of miRNA targeting sequences [465,467]. Oncolytic and immunogenic efficacy can be increased by insertion of certain transgenes encoding (i) enzymes that convert pro-drugs to cytotoxic products (e.g., HSV-TK or cytosine deaminase), (ii) immunostimulatory cytokines (e.g., GM-CSF or IL-12), or (iii) TME/ECM-modifying peptides and enzymes (e.g., MMP-9 or the anti-angiogenic peptide angiostatin) [468]. Safety can be ensured by mutations in pathogenic and virulence genes as well as in genes required for viral replication in normal cells [457,468].

Nowadays, a large repertoire of oncolytic viruses is available and oncolytic virotherapy has been intensively investigated in numerous preclinical and clinical studies, also in combination with other cancer therapies like chemotherapy, radiation therapy, or other immunotherapies [456,457,458,459,460,469,470]. Table 2 displays approved oncolytic virotherapies and those that have been or are currently tested in clinical trials.

RIGVIR^®^ was the first oncolytic virus being approved for therapy of melanoma in Latvia in 2004 [471]. This oncolytic virus, enteric cytopathogenic human orphan (ECHO)-7, is a wild-type virus. In 2005, the first genetically modified oncolytic virus (Oncorine^®^, a recombinant oncolytic adenovirus H101) was approved in China for the treatment of nasopharyngeal carcinoma [474,475]. Ten years later, T-Vec (talminogene laherparepvec) achieved approval by the FDA and the European Medicines Agency (EMA) for treatment of advanced melanoma [477,478]. This oncolytic virus is derived from HSV-1 and was genetically modified to mitigate pathogenicity as well as to increase tumor-selective replication and lysis [478]. In addition, T-Vec expresses GM-CSF to enhance anti-tumor immunity.

Saha et al. conducted a preclinical study with a triple-mutated third generation oncolytic HSV-1 vector (G47Δ-mIL12), in which the murine IL-12 gene was inserted [483]. This oncolytic virus was applied intratumorally, in combination with systemically applied immune checkpoint inhibitors. Only the triple combination of G47Δ-mIL12, anti-CTLA-4, and anti-PD-1 antibodies successfully cured glioblastoma in an immune-competent glioblastoma mouse model.

Other oncolytic DNA viruses that are frequently used are genetically engineered adenovirus and vaccinia viruses [484,485]. CG0070, an oncolytic adenovirus type 5 with an inserted GM-CSF gene, is currently investigated in advanced clinical trials for treatment of non-muscle invasive bladder cancer (BOND, NCT01438112; BOND2, NCT02365818) [456]. The BOND study (phase II/III clinical trial) demonstrated that intravesically applied CG0070 evoked a durable response in a subset of high-risk patients and was well tolerated [476]. An example for an oncolytic vaccinia virus in clinical studies is pexastimogene devacirepvec (Pexa-Vec, JX-594), which bears a mutation in the TK gene for cancer cell targeting and an inserted GM-CSF gene to enhance immune stimulation [456,482,486,487,488]. In a phase III clinical trial, Pexa-Vec is currently evaluated in combination with the multi tyrosine kinase inhibitor sorafenib in patients with advanced hepatocellular carcinoma without prior systemic therapy [482].

Reolysin^®^ (pelareorep) is a wild-type oncolytic RNA virus (type 3 Dearing (T3D) strain reovirus) [457,489], which is extensively studied in clinical trials [456,472]. In phase II and III clinical trials, Reolysin^®^ showed encouraging clinical efficacy, especially in combination with chemotherapeutics (e.g., carboplatin and paclitaxel) in patients with advanced malignancies [472,473].

Despite rapid progress in oncolytic virotherapy and encouraging results in clinical trials, there are still some obstacles [457]. One shortcoming is the small genomic capacity of some oncolytic viruses [460]. Moreover, deletion of pathogenic genes to reduce toxicity might also reduce oncolytic activity [490]. Efficacy may be enhanced for instance by insertion of transgenes or combination with other therapies. In case of the latter, optimal therapy regimens and schedules have to be evaluated in terms of dosage, application routes, and timing [457]. Therefore, further investigations in clinical trials are needed.

### 5.4. Nucleic Acid-Based TLR Agonists to Boost Anti-Tumor Immune Response

PAMPs and other danger signals are recognized by the innate immune system via pattern recognition receptors such as TLRs [491,492]. Subsequently, pro-inflammatory pathways and the innate immune system are activated to eradicate pathogens. The anti-tumor immune response can be augmented by mimicking PAMPs. Monophosphoryl lipid A, a modified lipopolysaccharide derivative that triggers TLR4, is used as the adjuvant component in the prophylactic cervix cancer vaccine Cervarix^®^ [493]. The successful application of this TLR ligand also reinforced further research in immunostimulatory nucleic acids like double-stranded RNA (dsRNA) or single-stranded DNA (ssDNA) for cancer immunotherapy.

Poly(I:C) is an artificial dsRNA analog that acts as a potent TLR3 agonist [491,494]. Besides enhancement of the anti-tumor immune response, mainly by induction of IFN type I and chemokines especially in immune cells, poly(I:C) also directly induces apoptosis in cancer cells [495,496,497]. However, early clinical trials conducted in the 1970s using poly(I:C) for cancer treatment did not prove any clinical benefit [491,498,499,500], most likely because of its fast degradation prior to cellular uptake [501,502]. Consequently, a stabilized version of poly(I:C), polyriboinosinic:polyribocytidylic acid-polylysine carboxymethylcellulose (poly-ICLC, Hiltonol^®^), has been developed [502,503]. However, toxicity was a big issue in early rounds of clinical testing, which could be reduced by administration of lower intravenous doses and by local application [491]. Nowadays, poly-ICLC is intensively evaluated in phase I and II clinical trials, especially in combination with cancer vaccines and radiotherapy [491,492,495,504,505]. Another concept to increase the stability and to improve the toxicity profile of TLR3 agonists is the employment of particulate formulations [506]. Shir et al. designed poly(I:C) polyplexes using a polymer conjugate consisting of branched PEI, PEG, EGF for EGFR-targeting, and lytic melittin for improved endosomal escape [507,508]. Complete tumor elimination could be achieved via intratumoral application in three different tumor mouse models (glioblastoma, breast cancer, adenocarcinoma) [507], and in a disseminated EGFR overexpressing tumor mouse model [508]. In the latter study, polyplexes were administered intravenously, followed by intraperitoneal injection of peripheral blood mononuclear cells into tumor bearing immune-deficient SCID mice. Tumor-targeted poly(I:C) mediated induction of chemokines and inflammatory cytokines selectively within the tumor tissue. This led to tumor homing of the injected immune cells as well as a strong anti-tumor and bystander killing effect. The latter might be advantageous in view of the heterogeneous tumor tissue. In this study, complete curation was achieved without adverse side effects [508]. Schaffert et al. optimized the nano-carrier by using linear instead of branched PEI [509]. The improved carrier was effective even without the lytic melittin unit. In a follow-up study, GE11 peptide was used for EGFR targeting [510]. In contrast to EGF, GE11 does not activate EGFR, and thus mitogenic activity of the tumor cells should be much lower. This could be an advantage in terms of clinical use. Other types of poly(I:C) polyplexes were formulated by Lächelt et al. [511] using sequence-defined oligo(ethanamino)amides modified with PEG and the anti-folate drug methotrexate (MTX) with varying degrees of polyglutamylation. MTX exhibits dual function by serving as ligand targeting the folate receptor and by exerting cytotoxic effects in the cytosol. The extent of polyplex uptake as well as MTX and poly(I:C) toxicities correlated with increasing amounts of glutamic acid. A synergism of the combined cytotoxic agents was observed.

CpG ODNs are another class of TLR agonists that imitate bacterial/viral genomic sequences, and are recognized by TLR9 trough their unmethylated cytosine-guanine dinucleotide motif [87,512,513,514]. TLR9 signaling results in the secretion of pro-inflammatory cytokines and the activation of APC and CTL. To improve the in vivo stability of CpG ODNs in most cases the phosphodiester backbone is replaced (at least in part) by a nuclease-resistant phosphorothioate backbone [513,515]. Encouraging results in preclinical studies led to a series of clinical trials in the mid-2000s, testing CpG ODNs alone, in combination with cancer vaccines, or with chemo- and radiotherapy [18,514,515]. However, the clinical outcome fell far short of the hopes and expectations, especially in case of CpG ODN monotherapies, but safety and good tolerability were proven. Subsequent studies showed that TLR9 signaling was negatively influenced at several levels by the immunosuppressive TME [514]. Consequently, CpG ODNs in combination with immune checkpoint inhibitors are currently evaluated in phase I and II clinical trials for treatment of advanced solid tumors like metastatic melanoma [514]. Another dual immunotherapy strategy are conjugates of CpG ODN and either STAT3 siRNA or a STAT3 decoy ODN, respectively [514,516], as STAT3 is an oncogenic transcription factor that interferes with TLR9 signaling. Furthermore, NPs that deliver CpG ODN are under intensive investigation in several preclinical and also some clinical studies [513]. The goal of all these NP-based approaches is to enhance the therapeutic efficacy of CpG ODNs by increasing their stability and protection against nucleases as well as to improve the uptake of CpG ODNs by target cells. In addition, NPs allow to use phosphodiesters instead of the commonly used phosphorothioate backbone [513]. This may improve safety, as phosphorothioates are known to cause various adverse effects, especially in case of systemic application at higher doses [513,514]. By now, several types of NPs have been evaluated for CpG ODN delivery [18,513]. Preclinical studies are conducted inter alia with polymeric NPs formed with polymers like poly(lactic-co-glycolic acid) or PEI [517,518], liposomal formulations [519,520,521], carbon nanotubes [522,523], gold [524,525], and silica mesoporous NPs [526], as well as DNA-based carriers [527,528]. Near-infrared light responsive nanomaterials like copper sulfide, graphene oxide, or gold nanorods can be used for photothermal enhancing of CpG ODN immunogenicity [529,530,531]. Besides these preclinical studies, CpG ODN-loaded virus-like particles are already investigated in a phase I/II clinical trial [532]. Furthermore, CpG ODNs can also be conjugated with antigen (peptide/protein) or human immunodeficiency virus-derived Tat-peptide [533,534]. Self-assembled CpG ODNs like MGN1703 are another example, already tested in phase I and II clinical trials, for treatment of e.g., metastatic colorectal carcinomas [535,536,537].

### 5.5. Tumor Suppression by RNA Interference

The discovery of RNAi in 1998 [538] led to a better understanding of gene regulation mechanisms [103]. RNAi in humans and animals is mediated by miRNA [539] (Figure 2). miRNAs influence many cellular functions like proliferation, differentiation, apoptosis, oncogenesis, and drug sensitivity [540,541,542,543]. Dysregulated miRNA expression is associated with the development and progress of various diseases [539,543,544]. Calin et al. were the first to report involvement of miRNA in cancer progression [545]. miRNAs can be used as diagnostic and prognostic biomarkers [546]. For cancer therapy, oncogenic miRNAs can be blocked by antisense molecules (antagomirs), while attenuated levels of tumor suppressor miRNAs can be substituted by pre-miRNAs or miRNA mimics [539,543,547,548].

The major challenge in clinical translation of miRNA therapeutics is to ensure their efficient, specific, and safe delivery to the tumor [539,543,546,549]. Chemical modifications can increase resistance of RNA to enzymatic degradation by nucleases. Examples for such structural alterations are modifications of the ribose 2′-OH group, the use of phosphorothioate instead of phosphodiester bonds, peptide nucleic acids, locked nucleic acids as well as conjugation with other moieties (e.g., cholesterol, antibodies, or membrane translocation peptides) [103,539,550,551,552].

For example, Cheng et al. conjugated peptide nucleic acid-based anti-miR-155 to a pH-sensitive membrane translocation peptide via a disulfide link [103,552]. In the acidic tumor tissue, the conformational change in this peptide promoted internalization of the antagomir, which was released intracellularly upon disulfide cleavage due to increased glutathione levels. In a lymphoma model, cell targeting, a significant inhibition of lymphoma proliferation as well as a good tolerability were demonstrated. It is also worth noting that the neutral charge of the peptide nucleic acid was decisive for success.

Viral as well as non-viral delivery systems such as liposomal or polymeric NPs are under investigation to prevent degradation of miRNA and to promote their targeted delivery [539,546,551]. Loss of miR-200c expression is known to promote tumorigenic processes like tumor cell proliferation, EMT, migration, and chemoresistance [539,553,554,555,556,557,558]. Müller et al. tested a cationic oligo(ethanamino)amide structure with T-shape topology, terminal cysteines, and a dioleyl motif, post-functionalized with PEG-GE11 for shielding and EGFR targeting for delivery of a mimic of the tumor suppressor miR-200c [559]. In two different human tumor cell lines, these EGFR-targeting miRNA polyplexes conferred selective, enhanced delivery of miRNA-200c, leading to various anti-tumor effects, including decreased tumor cell proliferation and migration, and enhanced sensitivity towards doxorubicin.

Altogether, miRNA therapeutics hold great potential for efficient and safe cancer treatment, especially as multi-functional nano formulations, paving the way towards clinical translation. A liposomal formulation of miR-34a mimic (MRX34) for treatment of patients with advanced solid tumors was the first miRNA therapeutic entering phase I clinical studies in 2013, but was accompanied by severe immune-mediated adverse effects (NCT01829971) [560,561]. Nevertheless, the observed dose-dependent modulation of relevant target gene expression provided a proof-of-concept for miRNA-based cancer therapy [561]. This raises hope that miRNA therapeutics will make the leap towards clinical application. Therefore, further optimization of cargo and delivery systems to improve clinical efficacy and toxicity profiles is necessary.

## 6. Conclusions

Until a few years ago, nucleic acid-based immunotherapeutics have proven successful in preclinical studies, but largely fell short of expectations when evaluated for therapeutic efficacy in clinical trials [57,61]. One major limit has been the lack of appropriate delivery systems required to prevent degradation of pDNA/mRNA, and to enable cell type-specific delivery [125,126]. Insofar, it is not surprising that by now virus-based gene therapies including oncolytic viruses [471,474,475,477,478], and cell-based immunotherapeutics, namely CAR-T cell therapies [28,277,562,563,564], demonstrated more successful for tumor therapy, and have been approved for clinical treatment. However, in the last years, the development of biocompatible, cell targeting NPs, especially of liposomal carriers [565], has strongly improved the efficacy of e.g., mRNA-based anti-tumor vaccines [71,72]. Additionally, in case of CAR-T as an ex vivo gene therapy approach non-viral delivery is currently tested [566]. These developments, in combination with structural improvements in particular of gene encoding pDNA [567], and the proper choice of individual tumor-specific neoantigens for individualized vaccination [79], are important factors to overcome the low therapeutic efficacy of most nucleic acid immunotherapeutics tested so far. Furthermore, as numerous clinical trials have repetitively shown, nucleic acid-based therapeutics were more efficient when co-applied with agents that act on other levels like immune checkpoint inhibitors [70,79] or chemotherapeutics [474], and radiotherapy [568]. Moreover, first preclinical studies have shown that also different kinds of nucleic acids that act on distinct levels of cancer treatment may be combined to yield synergistic effects. For example, co-administration of the adjuvant poly(I:C) enhanced the anti-tumor efficacy of CAR-T cells [569]. Similarly, co-application of an oncolytic adenovirus and of CAR-T cells improved anti-tumor responses as compared to monotherapy [570].

Altogether, ongoing developments indicate that nucleic acid-based therapeutics will become essential tools for successful tumor therapy as part of combination therapies, comprising the induction of tumor antigen-specific immune reactions [79], the enhancement of anti-tumor responses [514], the inhibition or reprograming of regulatory immune cells [256], the generation of tumor killing immune cells (CAR) [284], and direct killing of tumor cells [482]. The versatility of nucleic acids as a therapeutic mean is underscored by the fact that these can exert either of the aforementioned functions by serving as gene expression units in pDNA/mRNA vaccines, conferring RNAi (siRNA, miRNA), and adjuvant activity (e.g., CpG ODN), and can be easily produced under GMP conditions [571]. Therefore, it is conceivable that in the future nucleic acid-based therapeutics that act on different levels of cancer treatment will be part of combination therapies involving either also conventional therapeutics or distinct types of nucleic acids.

## Figures and Tables

**Table 1:** Examples of clinical trials investigating nucleic acid-based approaches for adjusting intratumoral cytokine levels.

Signaling Molecule	Therapy Strategy	Application Route	Treated Cancer	Clinical State	References
IL-2	Syngeneic tumor cell vaccine modified with IL-2 gene ex vivo	Intradermal or subcutaneous injection	Metastatic melanoma	Phase I	[319]
	Allogeneic tumor cell vaccine modified with IL-2 gene ex vivo	Subcutaneous injection	Metastatic melanoma	Pilot study	[320]
				Phase I–II	[321]
	Allogeneic tumor cell vaccine modified with IL-2 gene ex vivo	Subcutaneous injection	Relapsed neuroblastoma	Phase I	[322]
TNF-α	TNFerade, a replication-deficient adenoviral vector encoding for TNF-α under the control of a radiation inducible promotor (erg-1 gene promotor)	Intratumoral injection	Various cancer types, e.g., advanced pancreatic cancer	Phase III	[323,324]
IL-12	Ad–RTS–hIL-12, an adenoviral vector encoding for IL-12 transgene designed with a ligand-inducible expression switch	Injection in the resection cavity	Recurrent high-grade glioma	Phase I	[325]
GM-CSF	GVAX, an allogeneic tumor cell vaccine modified with GM-CSF gene ex vivo,(in combination with immune checkpoint inhibitors and/or cyclophosphamide and Listeria monocytogenes-expressing mesothelin (CRS-207))	Intradermal injection	Advanced pancreatic cancer	Phase Ib	[326]
				Phase II	[327]
				Phase IIb	[328]
				Phase II	[329]
IFN-α	Instiladrin^®^ (rAdIFNα2b/Syn3), an IFN-α encoding adenoviral vector	Intravesical injection	BCG unresponsive bladder cancer	Phase III*—*results pending (NCT02773849)	[330]
TGF-β (inhibition)	Belagenpneumatucel-L, an allogeneic tumor cell vaccine altered to express ASO directed against TGF-β	Intradermal injection	Advanced non-small cell lung cancer	Phase II	[331,332]
				Phase III	[333]

**Table 2:** Examples of oncolytic virotherapies approved or in clinical trials.

Oncolytic Virus	Genetic Modification	Treated Cancer	Clinical State	Reference
Wild-Type Virus				
RIGVIR^®^ (wild-type ECHO-7; (+)ssRNA virus)	–	Melanoma	Approved in Lativa in 2004	[471]
Reolysin^®^ (pelareorep, type 3 Dearing (T3D) strain reovirus; dsRNA virus)	–	Many advanced malignancies (e.g., melanoma, sarcomas, non-small cell lung cancer, pancreatic adenocarcinoma)	Phase I and II	[457,472,473]
		Advanced, metastatic head and neck cancer	Phase III	[472]
Oncolytic Adenovirus (dsDNA virus)				
Oncorine^®^ (rAdV H101)	Deletion in E1B-55K and E3 genes	Nasopharyngeal carcinoma	Approved in China in 2005	[474,475]
CG0070 (AdV-5)	Deletion in E3 gene; insertion of GM-CSF gene	Non-muscle-invasive bladder cancer	Phase II/III (BOND, NCT01438112); phase II (BOND2, NCT02365818)	[456,476]
Oncolytic Herpes Simplex Virus, HSV-1 (dsDNA virus)				
T-Vec (talminogene laherparepvec)	Deletion in ICP34.5 and ICP47 genes; insertion of GM-CSF gene	Advanced melanoma	Approved by FDA and EMA in 2015	[477,478]
M032	Deletion in ICP34.5 gene; insertion of IL-12 gene	Glioblastoma multiforme	Phase I	[479]
G47Δ	Deletion in ICP34.5, ICP47 and ICP6 genes; insertion of GM-CSF gene	Recurrent glioblastoma, castration resistant prostate cancer, recurrent olfactory neuroblastoma	Clinical trials in Japan	[456,480,481]
Oncolytic Vaccinia Virus (dsDNA virus)				
Pexa-Vec (JX-594, pexastimogene devacirepvec)	Mutation in TK gene; insertion of GM-CSF gene	Advanced hepatocellular carcinoma	Phase III (in combination with sorafenib)	[482]

## Abbreviations

A2AR: adenosine A2A receptor; ALL: acute lymphoblastic leukemia; AML: acute myeloid leukemia; APC: antigen presenting cell; ASO: antisense oligonucleotide; AVET: adenovirus-enhanced transferrinfection; CAR: chimeric antigen receptor; BCG: Bacillus Calmette–Guerin; CAF: cancer-associated fibroblast; cAMP: cyclic adenosine monophosphate; CCL: C-C motif chemokine ligand; CLL: chronic lymphocytic leukemia; CLR: C-type lectin receptor; CPP: cell penetrating peptide; CEA: carcinoembryonic antigen; CSC: cancer stem cell; CTL: cytotoxic T lymphocyte; CTLA-4: CTL-associated protein 4; CXCL12: C-X-C motif chemokine 12; CXCR4: C-X-C motif chemokine receptor 4; DC: dendritic cell; DOPE: dioleoyl phosphatidylethanolamine; DOTAP: N-[1-(2,3-dioleoyloxy)propyl]-N,N,N-trimethylammonium chloride; DOTMA: 1,2-di-O-octadecenyl-3-trimethylammonium propane; DSPE: distearoylphosphatidylethanolamine; dsRNA: double-stranded RNA; ECHO-7: enteric cytopathogenic human orphan 7; ECM: extracellular matrix; EGFR: epidermal growth factor receptor; EMA: European Medicines Agency; EMT: epithelial–mesenchymal transition; ENO1: α-enolase; EPR: enhanced permeation and retention; FDA: united states food and drug administration; GM-CSF: granulocyte macrophage colony-stimulating factor; HD: hexanediol diacrylate; HSV: herpes simplex virus; IFN-α: interferon alpha; IL: interleukin; IRAK1: IL-1 receptor-associated kinase; JNK: c-Jun N-terminal kinase; KC: Kupffer cell; mcDNA: mini-circle DNA; LAMP: lysosomal-associated membrane protein; LSEC: liver sinusoidal endothelial cell; MHC: major histocompatibility complex; MDSC: myeloid-derived suppressor cell; MMP: matrix metalloprotease; MTX: methotrexate; NK: natural killer cell; NLS: nuclear localization signal; NP: nanoparticle; NHL: non-Hodgkin lymphoma; ODN: oligodeoxynucleotides; OEI: oligoethylenimine; PD-1: programmed cell death protein 1; PD-L1: PD ligand 1; PEG: polyethylene glycol; PEI: polyethylenimine; Pexa-Vec: pexastimogene devacirepvec; PLGA: poly-D,L-lactic-co-glycolic acid; Poly(I:C): polyriboinosinic:polyribocytidylic acid; poly-ICLC: polyriboinosinic:polyribocytidylic acid-polylysine carboxymethylcellulose; RNAi: RNA interference; ROS: reactive oxygen species; SCF: stem cell factor; scFv: single chain fragment variable; ssDNA: single-stranded DNA; STAT: signal transducer and activator of transcription; STING: stimulator of IFN genes; SV40: simian virus 40; T-Vec: talminogene laherparepvec; T3D: type 3 Dearing; TAA: tumor-associated antigen; TAM: tumor-associated macrophage; TCR: T cell receptor; Teff: effector T cell; TGF-β: transforming growth factor beta; Th: T helper cell; TIL: tumor-infiltrating lymphocyte; TK: thymidine kinase; TLR: toll-like receptor; TNF-α: tumor necrosis factor alpha; TRAF6: tumor necrosis factor receptor-associated factor 6; Treg: regulatory T cell; iTreg: induced Treg; tTreg: thymic Treg; VEGF: vascular endothelial growth factor; VEGFR: VEGF receptor; WT1: Wilms’ tumor 1.
